# Resilience mediates the association between self-harm and suicidal ideation in Chinese left-behind children

**DOI:** 10.1186/s12889-021-12153-1

**Published:** 2021-11-09

**Authors:** Yuanyuan Xiao, Fang Liu, Hailiang Ran, Wenhang Deng, Yusan Che, Die Fang, Ahouanse Roland Donald

**Affiliations:** 1 Department of Epidemiology and Health Statistics, School of Public Health, Kunming Medical University, Kunming, 650500 Yunnan China; 2 The First Affiliated Hospital, Kunming Medical University, Kunming, Yunnan China

**Keywords:** Self-harm, Suicidal ideation, Resilience, Mediation, Left-behind children

## Abstract

**Background:**

A significant association between self-harm (SH) and suicide ideation (SI) has been found in Chinese left-behind children (LBC). Existing literature suggests that resilience might be a mediator in this association. However, this hypothesis has not been effectively discussed. The major aim of our study is to analyze the possible mediation of resilience in SH-SI association in Chinese LBC.

**Methods:**

A population-based clustering sampling survey of 2619 LBC was conducted in southwestern China Yunnan province. Self-developed structured questionnaire was used to collect relevant information. Univariate and multivariate Logistic regression models were applied to estimate the associations between SH and SI, resilience and SI, and SH and resilience. Path analysis was adopted to measure the mediation of resilience, as well as its 5 dimensions, in the association between SH and SI. A subgroup analysis was further done to explore the mediation of resilience in the associations between SH severity and SI, SH repetition and SI, among self-harmed LBC.

**Results:**

Compared with LBC who reported no SH behaviors, the odds ratio (OR) for SI was 3.37 (95% CI: 2.63–4.31) among self-harmed LBC. Based on the path model, resilience significantly mediated a quarter of the total association between SH and SI. Among the 5 dimensions of resilience, emotion regulation, interpersonal assistance, and family support were the strongest mediators. Subgroup analysis revealed that, the mediation of resilience was only significant for SH severity and SI.

**Conclusions:**

Resilience played as a prominent mediator in SH-SI association among Chinese LBC. Resilience-centered intervention measures could be considered to reduce suicidal risk of this disadvantageous group.

## Background

There are approximately 69 million left-behind children (LBC) in China, which equivalent to 30% of the children in rural area [1]. LBC is generally defined as young person under the age of 16 whose parents, or one parent, goes out to work, and the other has no ability for guardianship [2]. The well-being of this expanded disadvantageous population has always been national and international focus. Compared with non-left-behind children (NLBC), LBC generally reported inferior physical and mental health status, like retarded body development [3], severer nutrient deficiency [4], and elevated risk of depression [5]. Moreover, two studies published in Chinese revealed that, the prevalence of self-harm (SH) was significantly higher in LBC than which in NLBC [6, 7]. The elevated risk of SH among Chinese LBC can hardly be called a surprise: although SH can occur at any age group, adolescent population is one of the most vulnerable [8], besides, it has been verified that incomplete family structure and dysfunctional family relationship can all contribute to increased risk of SH [9, 10].

SH is the strongest precursor of suicide: longitudinal evidence indicates that, self-harmers were observed an approximately 30-fold increase in risk of suicide, compared with the general population [11]. The most widely accepted theory of suicidality stresses on its continuum development, which starts from suicidal ideation (SI), through suicide plan and suicide attempt, ends at suicide completion [12]. From this perspective, the presence of SI can be seemed the harbinger of imminent suicidal behaviors. In our previous study, we found that SH and SI were prominently associated with each other in Chinese LBC: among LBC who had SH experience, 33.6% reported SI, about 3-fold of which in LBC who did not have SH history (11.4%) [13]. With this regard, understanding key factors involved in the intimate association between SH and SI is crucial for suicide prevention in Chinese LBC.

Resilience or psychological resilience is a topic of considerable study interest in the field of positive psychology. The American Psychological Association defines resilience as “the process of adapting well in the face of adversity, trauma, tragedy, threats or even significant sources of stress” [14]. A newly published meta-analysis by Dong et al. disclosed that, the resilience level of Chinese LBC was lower than NLBC [15]. We also corroborated this positive connection between SH and resilience in LBC previously: a higher level of resilience was associated with 60% reduction in SH odds [16]. Also, it has been suggested that resilience could be a protective factor for SI in other adolescent populations [17, 18]. Therefore, it is possible to suspect that resilience may play as a mediator or moderator in SH-SI association. Nevertheless, this assumption has not been thoroughly investigated in any Chinese adolescent populations, including LBC.

In the current study, we aimed to discuss the suspected mediation via resilience in the association between SH and SI among Chinese LBC. We put forward the following two assumptions: 1) Resilience shows prominent mediation in SH-SI association; 2) In self-harmed LBC, resilience further mediates the associations between SH severity and SI, SH repetition and SI.

## Method

### Study design and participants

A simple random clustering sampling strategy was applied to choose study participants from Guangnan city, Wenshan Zhuang and Miao Minorities Autonomous Region, Yunnan Province, China. From altogether 18 streets or townships within Guangnan’s jurisdiction, 3 were randomly sampled (Babao, Jiumo and Zhulin). In this study, we adopted the following criteria to define LBC: children under 18 years old, with one or both of the parents migrated to other places for work, and the separation exceeds a consecutive 6 months in the past year. Eligible participants within the chosen sites were preliminarily included as potential participants. Considering that children cannot totally understand the definition and consequence of suicide until 10 years old [19], we only included potential participants who aged above 10. The following exclusion criteria were further applied: 1) Illiterate; 2) Unconscious or mentally ill; 3) Auditory dysfunction or language dysfunction; 4) Refuse to participate.

The survey was conducted between June 26 and July 6, 2018. Face-to-face interview method was used to collect relevant information from all eligible participants. Interviewers were all pre-trained medical students, who were either senior undergraduates or graduates majoring in clinical medicine or public health. The questionnaire was self-developed and contains 5 modules, which measuring general characteristics, SH behaviors, depressive symptoms, resilience, and SI, respectively. Before interview, written and oral consents were obtained from the legal guardians and the participants. Prior to the survey, a pre-survey had been done for 269 LBC chosen from a single school at the study site, and the results of this pre-survey indicated satisfactory internal validity for major instruments used in this study [13]. The study protocol was reviewed and approved by the Ethics Review Board of Kunming Medical University.

### Measurements and definitions

The Modified version of Adolescents Self-harm Scale (MASHS) devised by Feng et al. was used to evaluate the 18 most commonly seen SH behaviors among the study participants [20]. MASHS not only measures lifetime occurrence of the specific types of SH, but also the frequencies of them. Resilience was gauged by using the Resilience Scale for Chinese Adolescents (RSCA) [21], a 27-item questionnaire which can be divided into 5 dimensions (goal concentration, emotion regulation, positive perception, family support, interpersonal assistance). The answers to each item were 5-point Likert style. A higher combined score of RSCA indicates a higher resilience level in general. Finally, for lifetime SI, the Beck Scale for Suicide Ideation (BSSI) was adopted [22]. Specifically, if the participants answered “Weak”/“Moderate to strong” to the question “Desire to make active suicide attempt”, or, answered “Would leave life/death to chance”/“Would avoid steps necessary to save or maintain life” to the question “Passive suicidal desire”, they were then deemed as suicide ideators. Depressive symptoms were evaluated by using the Chinese version of the Children’s Depression Inventory (CDI), with a recommended cut-off of 20 for Asian adolescents was used to discriminate depressive individuals [23].

### Statistical analysis

Descriptive statistics, together with corresponding statistical tests (such as *t* test, Chi-square test, rank-sum test), were used to describe and compare the major characteristics of the study subjects. Univariate and multivariate Logistic regression models were adopted to estimate the associations between SH and SI, SH and resilience, and resilience with SI. Specifically, for resilience, as there is no commonly recommended cut-off of RSCA, here in this study, in Logistic regression analysis, we chose to dichotomize study subjects based on the calculated median (94). Path analysis was applied to estimate the mediation of resilience in SH-SI association based on multivariate analysis results. A series of subgroup path analyses which only included self-harmed LBC was then performed to further evaluate the mediation of resilience in the associations between SH severity and SI, SH repetition and SI. To satisfy continuity requirement, resilience was incorporated into all path models as its original quantitative form.

All analyses were performed by using the R software (Version 3.3.3, The R Foundation for Statistical Computing, Vienna, Austria). Considering that the clustering sampling strategy that we used will inevitably cause uneven sampling probability, survey data related packages, such as “survey”, “lavaan.survey” were used consistently to adjust for standard errors of the estimations. The significance level was set as two-tailed probability less than 0.05, only with the exception for univariate Logistic regression models, which adopted a lower level of 0.10 to screen for potential covariates.

## Results

### General characteristics of the participants

A total of 3011 eligible LBC were initially approached, among them, 28 refused to participate, 10 were over-aged, 354 were further deleted because of missing information in critical analytical variables, therefore the final analysis of this study was based on 2619 participants, and the effective response rate was 87.0%. The general characteristics of the included participants were presented in Table 1. A total of 555 participants were identified as suicidal ideators, with the estimated lifetime SI prevalence of 21.20% (95% CI: 17.30–25.00%). Depression status, resilience, and SH behaviors were significantly different between SI and non-SI groups: SI group was found higher prevalence of depressive symptoms, lower resilience score, and severer SH behaviors.

**Table 1:** General characteristics of 2619 LBC, Guangnan, Yunnan, China, 2018

Characteristics	Combined (*N* = 2619)	SI (*N* = 555)	Non-SI (*N* = 2064)	Statistic (*p*)
Demographic				
Sex: Boys (*N*, %)	1342 (51.24)	241 (43.42)	1101 (53.34)	17.23 (< 0.01) ^b^
Age (Mean, SD)	14.01 (1.79)	14.13 (1.65)	13.98 (1.82)	0.83 (0.50) ^a^
Current education (*N*, %)				23.75 (< 0.01) ^b^
Primary school	652 (24.89)	98 (17.66)	554 (26.84)	
Junior middle school	1464 (55.90)	356 (64.14)	1108 (53.68)	
Senior middle school	503 (19.21)	101 (18.20)	402 (19.48)	
Ethnicity (*N*, %)				0.57 (0.21) ^b^
Zhuang or Miao	2018 (77.05)	421 (75.86)	1597 (77.37)	
Other	601 (22.95)	134 (24.14)	467 (22.63)	
Socioeconomic				
Father’s age (Mean, SD)	39.47 (5.43)	39.53 (5.37)	39.46 (5.45)	0.26 (0.84) ^a^
Mother’s age (Mean, SD)	37.77 (5.15)	37.86 (5.20)	37.75 (5.14)	2.87 (0.21) ^a^
Father’s education level (*N*, %)				1.60 (0.12) ^b^
Elementary or below	2157 (82.36)	447 (80.54)	1710 (82.85)	
Junior middle school or above	462 (17.64)	108 (19.46)	354 (17.15)	
Mother’s education level (*N*, %)				0.66 (0.28) ^b^
Elementary or below	2450 (93.55)	515 (92.79)	1935 (93.75)	
Junior middle school or above	169 (6.45)	40 (7.21)	129 (6.25)	
Depressive symptom: CDI score ≥ 20 (*N*, %)	217 (8.29)	115 (20.72)	102 (4.94)	143.30 (< 0.01) ^b^
Resilience (Median, IQR)				
Combined RSCA score	94 (17)	88.97 (17)	96.02 (16)	− 159.7 (< 0.01)^c^
Dimension 1: Goal concentration	19 (4)	19 (5)	20 (5)	−3.14 (0.20) ^c^
Dimension 2: Emotion regulation	19 (6)	17 (6.5)	20 (7)	−5.46 (0.12) ^c^
Dimension 3: Positive perception	15 (4)	15 (3)	15 (4)	−2.26 (0.27) ^c^
Dimension 4: Family support	22 (5)	20 (5)	22 (5)	−30.86 (0.02) ^c^
Dimension 5: Interpersonal assistance	21 (7)	19 (8)	21 (6)	−15.35 (0.04) ^c^
SH behaviors (*N*, %)				
Any SH	1269 (48.45)	410 (73.87)	859 (41.62)	182.20 (< 0.01) ^b^
Repeated SH	930 (35.03)	337 (60.72)	583 (28.25)	101.86 (< 0.01) ^b^
Medium and above SH (Severity)	335 (12.79)	138 (24.86)	197 (9.54)	92.03 (< 0.01) ^b^

^a^Design-based *t* test

^b^Pearson Chi-square test (with Rao & Scott adjustment)

^c^Design-based rank-sum test

### Associations between SH, resilience, and SI

A series of univariate and multivariate Logistic regression models have been fitted to discuss the associations between SH and SI, resilience and SI, SH and resilience, and the results were collectively displayed in Table 2. After adjusted for potential covariates, SH and resilience were significantly associated with SI: LBC who had ever self-harmed were observed 3.37 (95% CI: 2.63–4.31) folds of odds in SI; LBC with higher level of resilience, defined as RSCA≥94, were seen a 33% (95% CI: 23–42%) reduction in SI odds. The adjusted association between SH and resilience was also statistically prominent: compared with never self-harmed LBC, the adjusted odds ratio (OR) for a higher level of resilience (defined as RSCA≥94) was 0.46 (95% CI: 0.39–0.53) in self-harmed LBC.

**Table 2:** Univariate and multivariate Logistic regression fitting results for SH, resilience and SI associations

Covariates	SI (Event: Yes)		Resilience (Event: RSCA≥94)	
	Crude OR (90% CI)	Adjusted OR (95% CI)	Crude OR (90% CI)	Adjusted OR (95% CI)
Sex (Ref: Boys): Girls	1.49 (1.26, 1.76)	1.57 (1.26, 1.96)	0.92 (0.85, 0.99)	0.91 (0.86, 0.96)
Age: + 1 year	1.05 (0.96, 1.15)		1.07 (1.00, 1.13)	1.08 (0.98, 1.20)
Ethnicity (Ref: Other): Zhuang or Miao	0.94 (0.87, 1.02)		1.10 (1.02, 1.18)	1.12 (1.00, 1.24)
Current education (Ref: Primary school):				
Junior middle school	1.82 (1.68, 1.96)	1.58 (1.47, 1.70)	0.89 (0.84, 0.94)	0.88 (0.66, 1.17)
Senior middle school	1.42 (1.21, 1.66)	1.42 (1.26, 1.60)	1.34 (1.17, 1.54)	1.08 (0.57, 2.05)
Father’s age: + 5 years	1.01 (0.94, 1.09)		0.95 (0.89, 1.01)	
Mother’s age: + 5 years	1.02 (1.01, 1.03)	0.97 (0.89, 1.05)	0.99 (0.91, 1.06)	
Father’s education (Ref: Elementary or below)				
Junior middle school or above	1.12 (0.99, 1.25)		1.26 (1.03, 1.54)	1.22 (0.92, 1.62)
Mother’s education (Ref: Elementary or below)				
Junior middle school or above	1.11 (0.95, 1.31)		1.31 (1.18, 1.45)	1.31 (1.09, 1.59)
Depression (Ref: < 20): ≥20	5.03 (4.44, 5.70)	3.26 (3.08, 3.45)	0.11 (0.08, 0.15)	0.13 (0.08, 0.18)
Ever self-harmed (Ref: No): Yes	3.97 (3.35, 4.70)	3.37 (2.63, 4.31)	0.40 (0.35, 0.47)	0.46 (0.39, 0.53)
Resilience (Ref: RSCA< 94): RSCA≥94	0.44 (0.42, 0.45)	0.67 (0.58, 0.77)	NA	NA

### Mediation of resilience in SH-SI association

**Fig. 1:**
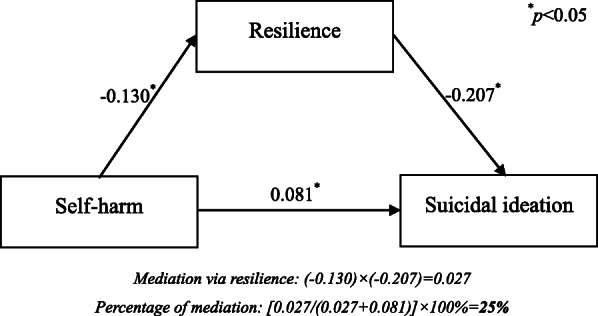
A path model illustrating the mediation of resilience in SH-SI association

Based on the positive findings from multivariate Logistic regression models, it is possible to suspect that resilience may mediate the association between SH and SI. Therefore, we constructed a hypothesized path model to validate this hypothesis. The structure and results of this path model were illustrated in Fig. 1: resilience presented significant mediation in SH-SI association, the standardized path coefficients between SH and resilience, resilience and SI were − 0.130 and − 0.207, and the mediation via resilience accounted for 25.0% of the total association between SH and SI.

**Fig. 2:**
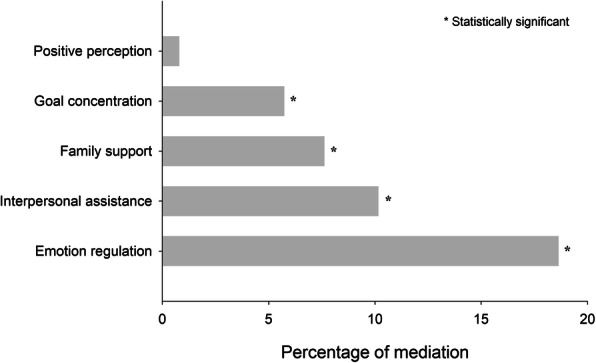
Percentages of mediation in the total SI-SH association by dimensions of resilience

We further dissected the mediation of resilience by using its 5 dimensions separately. Except for positive perception, the rest 4 dimensions all introduced significant mediation to SH-SI association. Emotion regulation was the strongest mediator, accounted for 18.6% of the total association, followed by interpersonal assistance (10.2%) and family support (7.6%) (Fig. 2).

### Subgroup analysis

**Fig. 3:**
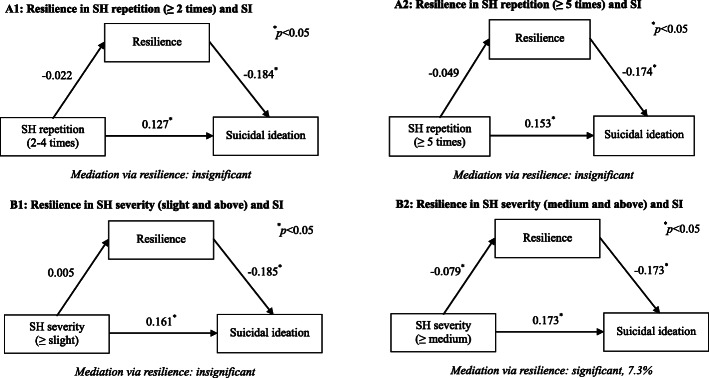
Path models for mediation via resilience in the associations between SH repetition and SI, SH severity and SI. Panel A1: the reference for SH repetition is “no repetition”; Panel A2: the reference for SH repetition is “4 times and below”; Panel B1: the reference for SH severity is “non-observable”; Panel B1: the reference for SH severity is “slight and below”

To analyze the possible mediation of resilience in the associations between SH severity and SI, SH repetition and SI, we performed a series of path analysis in self-harmed LBC only. The results were shown in Fig. 3. No matter what cut-offs had been chosen to define SH repetition (≥ 2 times or ≥ 5 times), resilience only presented insignificant mediation in the association between SH repetition and SI. However, for LBC who had committed more serious SH, a prominent mediation via resilience has been identified, which accounted for 7.3% of the total association between SH severity and SI.

## Discussion

In this cross-sectional study with a large representative sample of 2619 Chinese LBC, we evaluated suspicious mediation introduced by resilience in the association between SH and SI. The analytical results were supportive to the hypothesis that we put forwarded previously: resilience presented as a significant mediator in this association, contributed to one-fourth of the total SH-SI association. In our previously published studies, we have paid considerable attention to resilience and its association with SH behaviors among Chinese LBC. We found that, at first, resilience was inversely associated with SH, as a higher level of resilience has been found related to 0.40 folds of SH odds [16]. Besides, resilience may simultaneously play as a salient mediator in the association between depression and SH, as an estimated 26.8% of the total depression-SH association has been mediated via resilience [24]. Findings of the current study is an important extension on the pivotal role of resilience in suicide prevention and control among Chinese LBC: building-up resilience may not only be effective in antagonizing SH behaviors in the first place, but also can be beneficial for those who had already harmed themselves.

Among the 5 dimensions of resilience, emotion regulation presented as the strongest mediator in SH-SI association. Emotion regulation is the ability to respond to the ongoing demands of experience with the range of emotions in a tolerable manner [25]. The positive link between SH and emotion dysregulation has been repeatedly mentioned. In fact, it has been suggested that among adolescents, choosing SH is in essence an emotion regulation strategy [26]. In a previously published study on Hong Kong college undergraduates, the authors found that increased cognitive reappraisal, one of the two strategies of emotion regulation, was associated with reduced risk of suicidal behavior [27]. All these findings suggest the promising role of improving emotion regulation ability in reducing suicidal risk among Chinese LBC.

Interpersonal assistance and family support are the other two dimensions of resilience which presented noticeable mediation in SH-SI association. The two dimensions largely measure social support from different sources for LBC. Social support has been identified as a protective factor against suicidality among adolescents. For example, Matlin et al. revealed that, increased family support and peer support were associated with decreased suicidality for African American adolescents [28]. Besides, Miller et al. disclosed a prominent relationship between a lower support from close friends and higher odds of suicidal attempts [29]. In our previous study, we also identified that perceived social support from friends and parents were inversely associated with SI in LBC [30]. This prominent mediation of social support in SH-SI association also reminds us that, intervention measures which focusing on improving social support could simultaneously be considered in preventing SH associated suicidal risk among the Chinese LBC.

Emotion regulation more represents intrinsic ability-oriented effect, whereas social support more reflects circumstance influences. This heterogeneity in nature determines that for these two dimensions of resilience, intervention measures could be totally different. Interventions on the former should be targeted at individuals, while interventions on the latter should be focused on the environment, especially family and school for children and adolescents. Currently, various intervention measures aiming at enhancing emotion regulation ability of children and adolescents had been proposed and proven effective, like anger control skills [31], cognitive-behavioral therapies [32], methods of increasing tolerance of negative emotions [33], and more recently, the school-based Rochester Resilience Project developed by Wyman et al [34]. However, none of these intervention measures mentioned above has ever been implemented in Chinese children or adolescents, therefore their effectiveness in improving emotion regulation ability among Chinese LBC should be comprehensively evaluated.

For improving social support, environmental attributes should be considered. At the community level, it has been found that a comprehensive intervention strategy, which incorporated group psychotherapy and community support, was effective in improving perceived social support of elderly Chinese tuberculosis patients [35]. School-based intervention also showed promising effect. In a newly published randomized controlled trial on 60 Chinese college students, Li et al. summarized that group interpersonal psychotherapy (G-IPT), a structured short-term psychotherapy that stresses on resolving interpersonal problems, can effectively increase social support, and its intervention effect lasted long [36]. Family-based therapy could be adopted, too, under the premise that cultural difference has been considered. Chinese families are tied closely by blood relationship, and value family intimacy [37]. Under this situation, for both-parent-migrant LBC, the major caretakers are grandparents. In fact, it has been estimated that more than 70% Chinese LBC are under the guardianship of their grandparents [38]. Therefore, for both-parent-migrant LBC, family-based interventions should be more focused on cultivating the grandparent-grandchild cohesion. Nevertheless, as family-based intervention programs remain rare in mainland China, especially in rural areas, for Chinese LBC, community-based or school-based social support interventions maybe more applicable and resourceful, therefore should be implemented at first.

In self-harmed individuals, those who committed severer or repeated SH were of particularly increased suicidal risk [39, 40]. Therefore, we further analyzed the mediation of resilience in the associations between SH severity and SI, SH repetition and SI. One interesting finding is that the mediation of resilience was only significant for the association between severer SH and SI. As for the association between SH repetition and SI, resilience presented negligible mediation. This finding could suggest that, even for severer self-harmed LBC, resilience-based intervention measures may gain noticeable benefit in reducing suicide risk. The underlying mechanisms for this incongruent mediation of resilience should be further discussed by future studies.

Our study is the first exhaustive attempt in discussing the mediation of resilience in the association between SH and SI among Chinese LBC, a vulnerable population of elevated suicidal risk. Except for novelty and secular significance of the topic, scientific sampling design and elaborate statistical analysis further strengthened the validity of our results. Nevertheless, several limitations should be noticed. First, cross-sectional design undoubtedly prevents causal inference, therefore the positive findings based on path analysis are only possible mediation that should be corroborated by using longitudinal data. Second, self-report method in collecting relevant information may introduce information bias. Third, some potential confounders were not adjusted for because their information had not been collected, which can result in residual confounding. Finally, we only discussed the suspected mediation of resilience in SH-SI association, as the possibility of moderation by resilience also exists, this problem invites further investigation.

## Conclusion

To conclude, in this cross-sectional study of 2619 Chinese LBC, we found that resilience presented as a prominent mediator in the association between SH and SI. Among all dimensions of resilience, emotion regulation, interpersonal assistance and family support exhibited the strongest mediation. Our study results probably suggest resilience-centered intervention measures, especially those targeting at enhancing emotion regulation ability and improving social support, may exhibit effect in reducing SH associated suicidal risk in this expanded vulnerable population. Future studies of longitudinal design are needed to corroborate this hypothesis.

## Data Availability

The datasets used and/or analyzed during the current study are available from the corresponding author on reasonable request.

## Abbreviations

LBC: Left-behind children
SH: Self-harm
SI: Suicidal ideation
RSCA: The Resilience Scale for Chinese Adolescents
MASHS: The Modified version of Adolescents Self-Harm Scale
BSSI: The Beck Scale for Suicide Ideation
CDI: The Children’s Depression Inventory

## References

[1] UNICEF (2019). UNICEF China 2018 Annual Report.

[2] The State Council (2016). Opinions in strengthening care and protection of rural left-behind children.

[3] Zhang N, Becares L, Chandola T (2015). Does the timing of parental migration matter for child growth? A life course study on left-behind children in rural China. BMC Public Health.

[4] Luo J, Peng X, Zong R, Yao K, Hu R, Du Q (2008). The status of care and nutrition of 774 left-behind children in rural areas in China. Public Health Rep.

[5] He B, Fan J, Liu N, Li H, Wang Y, Williams J, Wong K (2012). Depression risk of ‘left-behind children’ in rural China. Psychiatry Res.

[6] Xu Y, Ma L (2013). A discussion on characteristics and causes of self-harm in rural left-behind teenagers: based on the survey in Macheng, Hubei province. J South-Central University for Nationalities (Humanities and Social Sciences).

[7] Xu Z, Su H, Wu G, Chang W, Sun Y (2010). Self-injurious behavior among rural stay-at-home middle school students and its relationship with internal and external locus of control. Chin J Public Health.

[8] Fliege H, Lee JR, Grimm A, Klapp BF (2009). Risk factors and correlates of deliberate self-harm behavior: a systematic review. J Psychosom Res.

[9] Sourander A, Aromaa M, Pihlakoski L, Haavisto A, Rautava P, Helenius H, Sillanpää M (2006). Early predictors of deliberate self-harm among adolescents. A prospective follow-up study from age 3 to age 15. J Affect Disord.

[10] Portzky G, De Wilde EJ, Van Heeringen K (2008). Deliberate self-harm in young people: differences in prevalence and risk factors between the Netherlands and Belgium. Eur Child Adolesc Psychiatry.

[11] Cooper J, Kapur N, Webb R, Lawlor M, Guthrie E, Mackway-Jones K, Appleby L (2005). Suicide after deliberate self-harm: a 4-year cohort study. Am J Psychiatry.

[12] Sveticic J, De Leo D (2012). The hypothesis of a continuum in suicidality: a discussion on its validity and practical implications. Ment Illn.

[13] Xiao Y, He L, Chang W, Zhang S, Wang R, Chen X, Li X, Wang Z, Risch HA (2020). Self-harm behaviors, suicidal ideation and associated factors among rural left-behind children in West China. Ann Epidemiol.

[14] American Psychological Association (2014). The road to resilience.

[15] Dong B, Yu D, Ren Q, Zhao D, Li J, Sun YH (2019). The resilience status of Chinese left-behind children in rural areas: a meta-analysis. Psychol Health Med.

[16] Tian X, Chang W, Meng Q, Chen Y, Yu Z, He L, Xiao Y (2019). Resilience and self-harm among left-behind children in Yunnan, China: a community-based survey. BMC Public Health.

[17] Kim SM, Kim HR, Min KJ, Yoo SK, Shin YC, Kim EJ, Jeon SW (2020). Resilience as a protective factor for suicidal ideation among Korean workers. Psychiatry Investig.

[18] Cheung VHM, Chan CY, Au RKC (2019). The influence of resilience and coping strategies on suicidal ideation among Chinese undergraduate freshmen in Hong Kong. Asia Pac Psychiatry.

[19] Mishara BL (1999). Conceptions of death and suicide in children ages 6-12 and their implications for suicide prevention. Suicide Life Threat Behav.

[20] Feng Y (2008). The relation of adolescents’ self-harm behaviors, individual emotion characteristics and family environment factors.

[21] Hu Y, Gan YQ (2008). Development and psychometric validity of the resilience scale for Chinese adolescents. Acta Psychol Sin.

[22] Beck AT, Steer RA, Ranieri WF (1988). Scale for suicide ideation: psychometric properties of a self-report version. J Clin Psychol.

[23] Bang YR, Park JH, Kim SH (2015). Cut-off scores of the children’s depression inventory for screening and rating severity in Korean adolescents. Psychiatry Investig.

[24] Xiao Y, He L, Chen Y, Wang Y, Chang W, Yu Z (2020). Depression and deliberate self-harm among Chinese left-behind adolescents: a dual role of resilience. Asian J Psychiatr.

[25] Cole PM, Michel MK, Teti LO (1994). The development of emotion regulation and dysregulation: a clinical perspective. Monogr Soc Res Child Dev.

[26] Mikolajczak M, Petrides KV, Hurry J (2009). Adolescents choosing self-harm as an emotion regulation strategy: the protective role of trait emotional intelligence. Br J Clin Psychol.

[27] Ong E, Thompson C (2019). The importance of coping and emotion regulation in the occurrence of suicidal behavior. Psychol Rep.

[28] Matlin SL, Molock SD, Tebes JK (2011). Suicidality and depression among African American adolescents: the role of family and peer support and community connectedness. Am J Orthop.

[29] Miller AB, Esposito-Smythers C, Leichtweis RN (2016). Role of social support in adolescent suicidal ideation and suicide attempts. J Adolesc Health.

[30] Xiao Y, Chen Y, Chang W, Pu Y, Chen X, Guo J, Li Y, Yin F (2020). Perceived social support and suicide ideation in Chinese rural left-behind children: a possible mediating role of depression. J Affect Disord.

[31] Lochman JE, Coie JD, Underwood MK, Terry R (1993). Effectiveness of a social relations intervention program for aggressive and non-aggressive, rejected children. J Consult Clin Psychol.

[32] Asarnow JR, Scott CV, Mintz J (2002). A combined cognitive-behavioral family education intervention for depression in children: a treatment development study. Cognit Ther Res.

[33] Katz LY, Cox BJ, Gunasekara S, Miller AL (2004). Feasibility of dialectical behavior therapy for suicidal adolescent inpatients. J Am Acad Child Adolesc Psychiatry.

[34] Wyman PA, Cross W, Brown CH, Yu Q, Tu X, Eberly S (2010). Intervention to strengthen emotional self-regulation in children with emerging mental health problems: proximal impact on school behavior. J Abnorm Child Psychol.

[35] Li X, Wang B, Tan D, Li M, Zhang D, Tang C, Cai X, Yan Y, Zhang S, Jin B, Yu S, Liang X, Chu Q, Xu Y (2018). Effectiveness of comprehensive social support interventions among elderly patients with tuberculosis in communities in China: a community-based trial. J Epidemiol Community Health.

[36] Li Y, Roslan SB, Ahmad NAB, Omar ZB, Zhang L (2019). Effectiveness of group interpersonal psychotherapy for decreasing aggression and increasing social support among Chinese university students: a randomized controlled study. J Affect Disord.

[37] Jiang X, Chaiwan S, Panuthai S, Cheng Y, Yin L, Li J (2002). Family support and self-care behavior of Chinese chronic obstructive pulmonary disease patients. Nurs Health Sci.

[38] China Women’s Federation (2013). National survey of left-behind children in rural areas. Chinese Women’s Movement.

[39] Bergen H, Hawton K, Waters K, Ness J, Cooper J, Steeg S, Kapur N (2012). How do methods of non-fatal self-harm relate to eventual suicide?. J Affect Disord.

[40] Zahl DL, Hawton K (2004). Repetition of deliberate self-harm and subsequent suicide risk: long-term follow-up study of 11 583 patients. Br J Psychiatry.

